# Differentiation of Human Glioblastoma Stem Cells as a Prospective Tool to Find New Biomarkers and Molecular Targets: A Proteomic and Lipidomic Approach

**DOI:** 10.3390/ijms27219517

**Published:** 2026-10-24

**Authors:** Ana M. Cardoso, Diana Serra, Cristina P. R. Xavier, João P. Monteiro, Catarina M. Morais, Isabel M. Nunes Correia, Maria do Rosário M. Domingues, M. Helena Vasconcelos, Amália S. Jurado, Maria C. Pedroso de Lima

**Affiliations:** 1 CIBB—Centre for Innovative Biomedicine and Biotechnology, CNC-UC—Center for Neuroscience and Cell Biology, University of Coimbra, 3004-504 Coimbra, Portugal; cardoso.ams@gmail.com (A.M.C.); diana.serra@euvg.pt (D.S.); catimendesmorais@gmail.com (C.M.M.); inunescorreia@gmail.com (I.M.N.C.); 2 CIVG—Vasco da Gama Research Center, EUVG-Vasco da Gama University School, 3020-210 Coimbra, Portugal; jpspmonteiro@yahoo.com; 3 i3S—Instituto de Investigação e Inovação em Saúde, Universidade do Porto, 4200-135 Porto, Portugal; cristina.xavier@iucs.cespu.pt (C.P.R.X.); hvasconcelos@ipatimup.pt (M.H.V.); 4 Associate Laboratory i4HB, Institute for Health and Bioeconomy, University Institute of Health Sciences—CESPU, 4585-116 Gandra, Portugal; 5 UCIBIO—Applied Molecular Biosciences Unit, Toxicologic Pathology Research Laboratory, University Institute of Health Sciences (1H-TOXRUN, IUCS-CESPU), 4585-116 Gandra, Portugal; 6 Centro de Espetrometria de Massa & LAQV-REQUIMTE & Departamento de Química, Universidade de Aveiro, Campus Universitário de Santiago, 3810-193 Aveiro, Portugal; mrd@ua.pt; 7 Institute for Interdisciplinary Research, University of Coimbra, 3030-789 Coimbra, Portugal; 8 CESAM & Departamento de Química, Universidade de Aveiro, Campus Universitário de Santiago, 3810-193 Aveiro, Portugal; 9 Department of Biological Sciences, Faculty of Pharmacy (FFUP), University of Porto, 4050-313 Porto, Portugal

**Keywords:** G144 glioblastoma stem cells, BMP4-induced differentiation, cell proliferation, invasion, senescence, tumour sphere formation, SPRY4 (sprouty RTK signalling antagonist 4) protein, ether lipids, liquid chromatography–mass spectrometry (LC–MS/MS), siRNA-mediated gene silencing

## Abstract

Although glioblastoma stem cells (GSCs) constitute a small subpopulation of this highly heterogeneous brain cancer, they are widely recognised as key drivers of tumour recurrence after surgical resection and resistance to radiotherapy and chemotherapy. Differentiation of GSCs has, therefore, emerged as a strategy to increase glioblastoma susceptibility to therapeutic and surgical interventions. Cellular and molecular heterogeneity of glioblastoma limits the effectiveness of any treatment that relies on single molecules identified as disease-specific biomarkers. In this context, lipidomic and proteomic profiles constitute a source of information that can be translated into the identification of clinically relevant diagnostic and prognostic biomarkers, and novel therapeutic targets towards eradication of this lethal disease. Our initial studies demonstrated that BMP4 induced differentiation of the human glioblastoma stem cells G144 with approximately a 30-fold increase in expression of the astrocytic marker GFAP, which was coupled with a reduction in cell proliferation (33% decrease in EDU incorporation), invasion (61% decrease) and tumoursphere growth (53% decrease in area), and enhanced senescence-associated β-galactosidase activity (5.9-fold increase). Lipidomic and proteomic analysis (using data from 3 and 4 independent experiments, respectively) was then performed, allowing the identification of specific lipids and proteins exclusively detected or predominant in stem or differentiated G 144 cells. Importantly, our findings, which revealed a 6-fold increase in GFAP expression upon downregulation of a specific protein detected only in stem cells (SPRY4), pave the way for the employment of the RNAi-mediated knockdown strategy as a mechanistically distinct alternative to the use of BMP4, and highlight this protein as a candidate regulator associated with the G144 stem-like phenotype.

## 1. Introduction

Glioblastoma (GB) is an extremely aggressive brain tumour that accounts for 50% of all malignant primary central nervous system (CNS) tumours [1], leading to death within a short period of time, with a median survival of approximately 15 months after diagnosis, even following surgical resection and standard treatment (radiotherapy plus temozolomide) [2].

Since innovative pharmacological therapies are commonly guided by the understanding of disease etiology, and the etiology of GB remains uncertain for the vast majority of patients, no curative treatment is currently available for GB, with radiotherapy and chemotherapy being used only for palliative care or life-extending purposes [1].

Regarding pathophysiology, GB is characterised by a highly complex cellular network, quite heterogeneous, comprising not only tumour glial cells but also a small population of stem cells (representing ~3% of the total cells within the tumour) [3]. Accumulated evidence shows that stem cells possess a self-renewal capacity, enabling them to proliferate indefinitely and, through mechanisms that are still unknown, drive tissue invasion [3], immune evasion [4], and vascular mimicry [5], which collectively contribute to the strong resistance of GB to anticancer agents. Therefore, the research community has been actively focused on characterising this stem cell subpopulation and its ability to initiate and propagate tumours, looking ahead to new targeted therapeutic approaches to overcome GB resistance and ultimately combat the disease [6,7].

The present study aimed to identify novel molecular targets to address current therapeutic challenges in GB. The rationale emerges from the fact that tumour recurrence and therapy resistance rely on GB stem cells residing in specialised and protective microenvironmental niches within the tumour mass [3]. Our perspective is that the efficacy of chemotherapeutics against GB could be enhanced by abrogating the tumourigenic capacity of the stem cell population through differentiation. The main hypothesis of our work is that the pernicious biological behaviour that distinguishes GB stem cells from differentiated cells is built upon distinct proteomic and lipidomic profiles, such that the uniqueness of certain protein and/or lipid components in GB stem cells will open doors to identifying prognostic biomarkers or innovative therapeutic targets.

By using bone morphogenetic protein 4 (BMP4) to induce stem cell differentiation, a deep molecular characterisation of human GB stem cells, encompassing proteomic and lipidomic profiles, was performed and compared to their differentiated counterparts.

BMP4 is a transforming growth factor-β (TGF-β) family member that has been used as a prognostic marker for glioma [8], with higher expression in low-grade gliomas than in high-grade gliomas [9]. A large number of studies have examined how BMP4-induced differentiation affects the stemness-associated functional properties of GB stem cells [7,10,11,12,13,14]. Given the considerable variability in BMP4 response reported across patient-derived GB cultures and GB stem cell lines, further modulated by differences in treatment protocols [7,10,12,13,14], G144 glioblastoma stem cells—a well-established primary human GB model pioneered by Pollard et al. [7]—were selected for this study to elucidate the effects of BMP4-induced differentiation on core biological characteristics.

Our data demonstrated that BMP4 is highly effective in inducing GB stem cell differentiation, reducing cell proliferation, invasion, and tumoursphere area while enhancing senescence-associated β-galactosidase (SA-β-gal) activity. Lipidomic analysis, in turn, indicates that certain lipid species are exclusively present in either stem or differentiated cells, or differ quantitatively between these cellular states, making them potential biomarkers for stemness and differentiation, respectively. The uneven distribution of ether lipids between the two cell groups, with the mostly unsaturated species prevailing in stem cells, together with significant differences in monounsaturated fatty acids (MUFA) and polyunsaturated fatty acids (PUFA) content in some diacylphospholipids, with higher levels of PUFA in stem cells, emerged as relevant lipid-pattern peculiarities of these cells. Therefore, a hypothesis-driven manipulation of the lipid profile of these cells, for example, through RNAi technology-based modulation of specific enzymes involved in lipid metabolism, may open avenues for innovative GB therapies by counteracting stem cell maintenance or promoting their differentiation.

Proteomic analysis allowed us to identify Sprouty RTK Signalling Antagonist 4 (SPRY4) as a new biomarker in GB stem cells and amenable to therapeutic gene silencing. Importantly, similar to BMP4-induced differentiation of G144 glioblastoma stem cells, SPRY4 silencing resulted in significantly higher expression of the GFAP astrocytic differentiation marker. These findings suggest that SPRY4 represents a relevant molecular target to promote GB stem cell differentiation and drive new pharmacological interventions to effectively tackle this aggressive malignancy.

## 2. Results

### 2.1. BMP4-Induced Differentiation of G144 Glioblastoma Stem Cells Decreases Cell Proliferation Rate, Enhances Senescence-Associated β-Galactosidase Activity and Reduces Invasion and Tumoursphere Size

Our initial studies addressed the biological effects of BMP4 treatment on G144 GB stem cells. We emphasise that all the studies addressed in this and the following sections were performed using two different growth conditions: standard conditions, i.e., in the presence of the growth factors EGF and FGF, referred to as a stem cell-adequate medium, and another one established with the withdrawal of growth factors and addition of BMP4. Therefore, the effects assigned to BMP4, here and elsewhere, should be interpreted as a simplification, as they are actually those that result from the withdrawal of growth factors along with BMP4 supplementation. Also, for simplification, we will refer to cells that were grown in the latter conditions as differentiated cells. The validity of this interpretation, however, will be addressed below, in the Discussion section.

**Figure 1:**
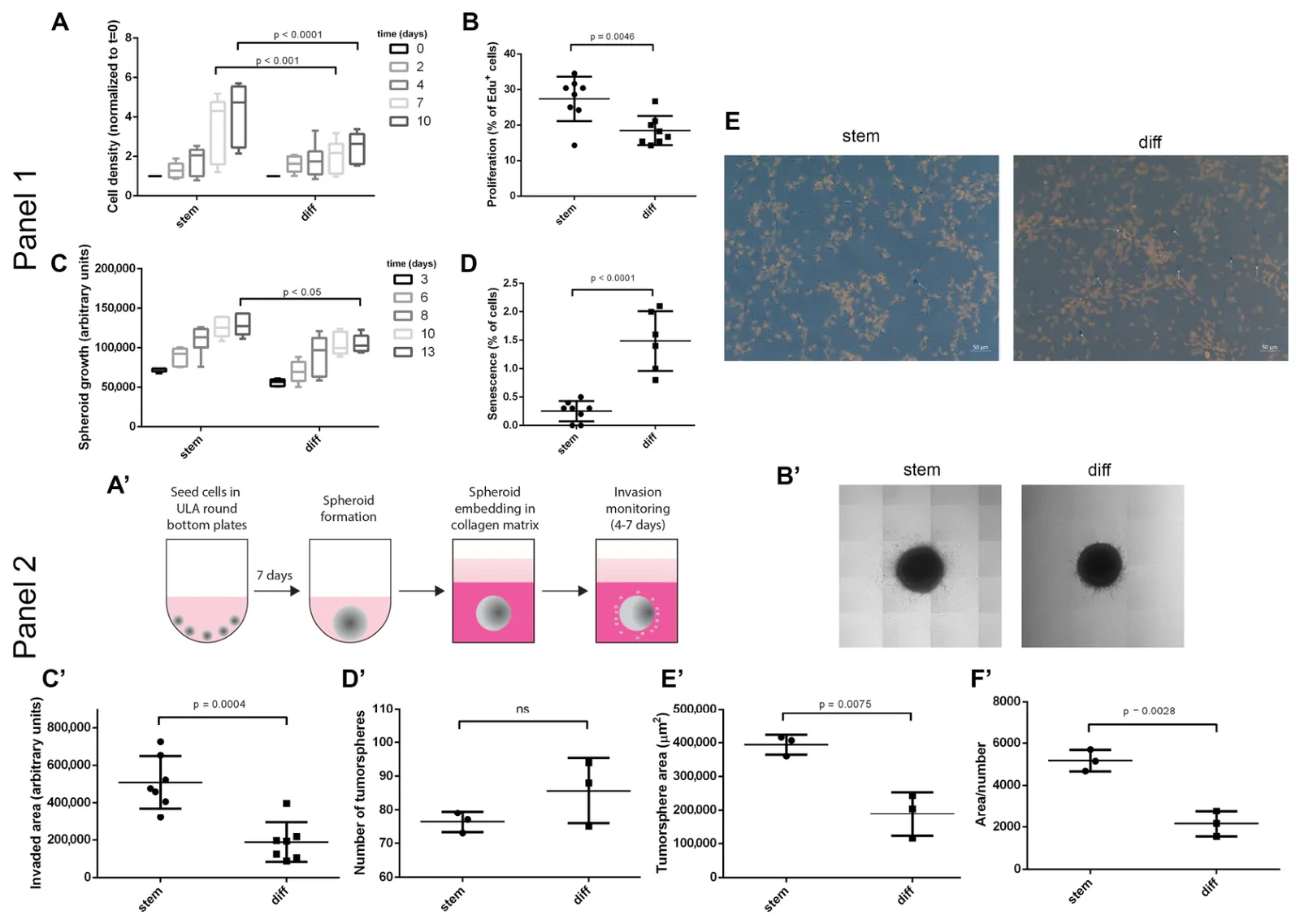
Effects of BMP4-induced differentiation on the proliferation and SA-β-gal activity (Panel 1) and on the invasion and tumoursphere formation (Panel 2) of G144 stem cells. Proliferation of stem and differentiated (diff) G144 cells, grown in monolayer cultures, was determined over time (**A**) by the SRB assay and by the EdU assay (**B**), with the proliferation rate evaluated as the percentage of EdU-positive cells after 24 h of cell incubation with EdU. The growth of spheroids prepared from G144 stem and differentiated cells was evaluated over time (**C**). β-galactosidase activity in G144 stem and differentiated cells was determined as the percentage of labelled blue (senescent) cells, with respect to the total number of cells per analysed microscopy field (**D**), as an indication of cell senescence. Representative fluorescence microscopy images (**E**) obtained from both stem and differentiated cells; senescent cells are stained blue and indicated with white arrows; cell nuclei are counterstained with Hoechst (orange). The experimental procedure for the cell invasion assay is schematically represented as a workflow from the formation of spheroids (a three-dimensional cell model) to the invasion monitoring (**A’**). The invasion areas (**C’**) for spheroids produced from both stem and differentiated cells were determined from images taken 3 days after spheroid embedding within the collagen matrix (**B’**), using a Carl Zeiss Axio Observer Z1 microscope equipped with a CCD digital camera (Axiocam HRm). The ability of G144 cells for tumoursphere formation was evaluated 4 days after growing in agarose, in terms of tumoursphere number (**D’**), tumoursphere total area (**E’**), and average area per tumoursphere (**F’**). Images of tumourspheres were obtained in an Axio Observer Z1 widefield microscope equipped with a CCD digital camera (Axiocam HRm).

Figure 1A clearly shows that the change of the cell culture medium, from a stem cell-adequate medium, which contains the growth factors EGF and FGF, to a medium lacking these growth factors but containing BMP4, added after 6 days of cell growth (see the descending arrow in Figure 1A), leads to a retardation of cell growth over time, as assessed by the sulforhodamine B (SRB) assay.

On the other hand, the determination of cell proliferation rate, through the 5-ethynyl-2′-deoxyuridine (EdU) assay (Figure 1B), shows that BMP4 induced a significant decrease in the EdU-positive cells after a 24 h incubation period in the presence of EdU. In fact, 18% of EdU-positive cells, with respect to the total number of cells, were observed when cells were grown in the presence of BMP4, as compared to 27% of EdU-positive cells when cells were grown in the stem cell adequate medium. To appreciate the effect of BMP4-induced cell differentiation in a tridimensional model, G144 stem and differentiated (diff) cells were cultured into spheroids and spheroid growth was monitored. As shown in Figure 1C, at the last time point, a significant decrease in cell growth was noticed for spheroids produced from differentiated cells as compared to those produced from stem cells. On the other hand, differentiation of G144 stem cells with BMP4 induced a very significant increase in SA-β-gal activity, as depicted in Figure 1D,E. Typical fluorescence microscopy images of stem and differentiated cells (Figure 1E) denote an increase in labelled blue cells (see white arrows) among differentiated cells, which results from 5-bromo-4-chloro-3-indolyl- β-D-galactopyranoside (X-gal) hydrolysis. In fact, β-galactosidase activity was found to be residual in stem cells and to increase six-fold in differentiated cells (Figure 1D).

To investigate the effect of BMP4-induced differentiation on the cell capacity for invasion, G144 stem cells and G144 differentiated cells were cultured over a 7-day period to form spheroids, which were then embedded into a collagen matrix, as depicted in Figure 1A’. At day 3 after embedding, cell invasion was assessed by quantifying the invasion areas (Figure 1C’) from microscopy images (Figure 1B’) of spheroids obtained from differentiated cells and from stem cells. As observed, at that time point, the area occupied by invading differentiated cells was significantly decreased compared to stem cells (Figure 1C’).

Since BMP4-induced differentiation reduced G144 stem cell proliferation and invasion competence, while increasing SA-β-gal activity, a step forward would be to prove that cell differentiation inhibits the generation of tumour-like structures. With this purpose, we examined the effect of BMP4-induced differentiation on tumoursphere formation. In fact, 4 days after growing in agarose, G144 stem cells formed colonies, termed tumourspheres, heterogeneous in size and shape. Although no significant difference was noticed regarding the number of tumourspheres formed by stem and differentiated cells (Figure 1D’), the total area occupied by the G144 stem cells was significantly larger than that occupied by differentiated cells (Figure 1E’). Consistently, the average area per tumoursphere was significantly larger for stem cells than for differentiated cells (Figure 1F’).

For Figure 1A,C, statistical analysis was performed using a two-way repeated-measures ANOVA, with cell type (stem vs. differentiated) and time as factors, followed by Sidak’s multiple comparisons test for comparisons between stem and differentiated cells at each time point. Adjusted *p* values are indicated in the graphs.

For the remaining graphs, pairwise comparisons were performed between stem and differentiated cells. All results are presented as mean ± SD of at least 3 independent experiments. The number of independent experiments was as follows: seven for each condition (stem and differentiated cells) in the invasion assay; eight for stem cells and six for differentiated cells in the senescence assay; and three for each condition (stem and differentiated cells) in the tumoursphere assay. Exact *p* values are reported in the graphs whenever available.

### 2.2. BMP4-Induced Differentiation of G144 Glioblastoma Stem Cells Promotes Significant Alterations in the Lipidomic Pattern

**Figure 2:**
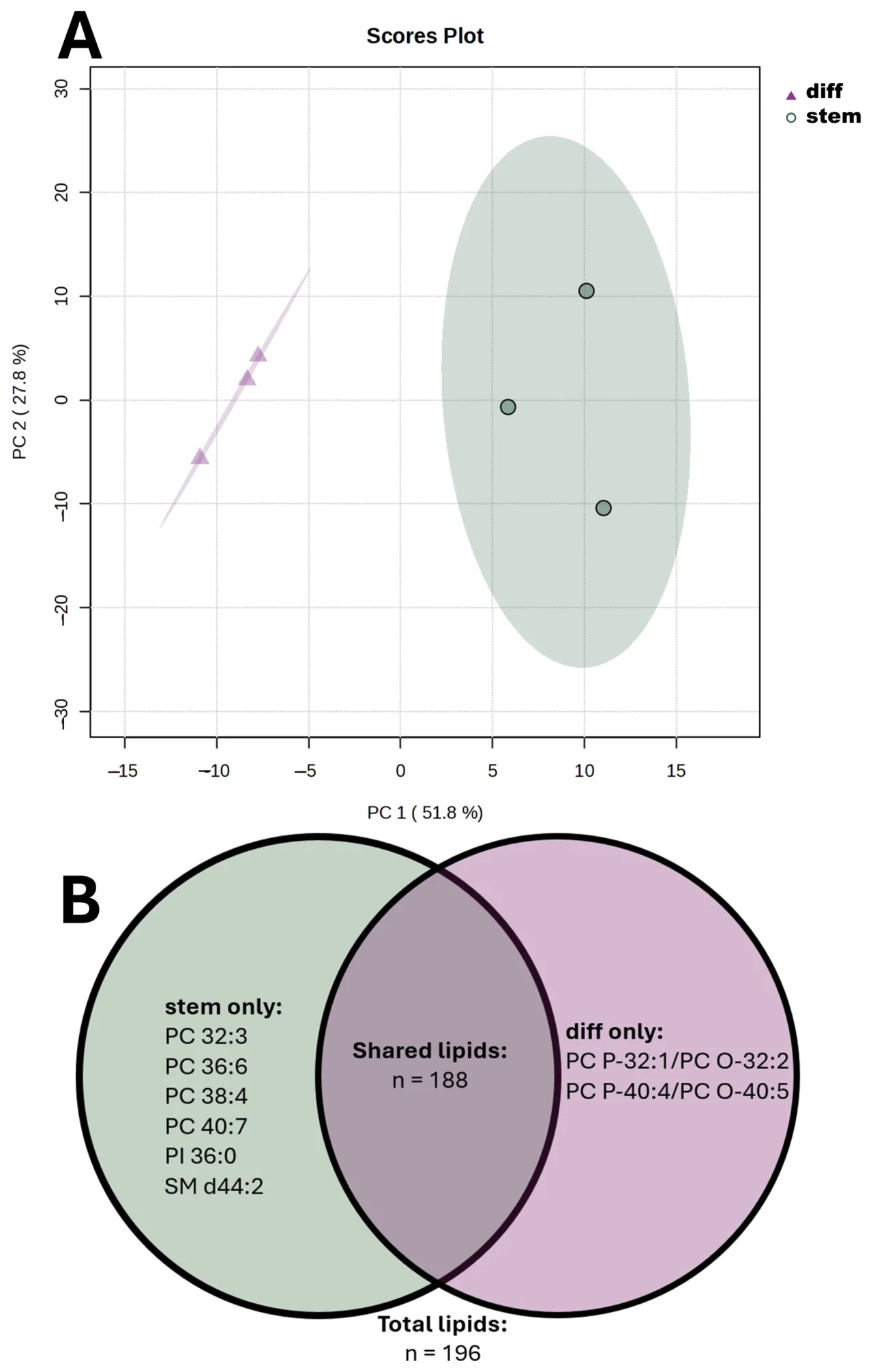
Lipidomic analysis of G144 stem and differentiated (diff) cells performed by liquid chromatography–mass spectrometry (LC–MS/MS). (**A**) Principal component analysis (PCA) of the data collected from the semi-quantitation of the detected lipid species; (**B**) Venn diagram depicting the differences in the lipid species detected between groups. Experimental data are the result of the analysis of 3 independent experiments.

**Figure 3:**
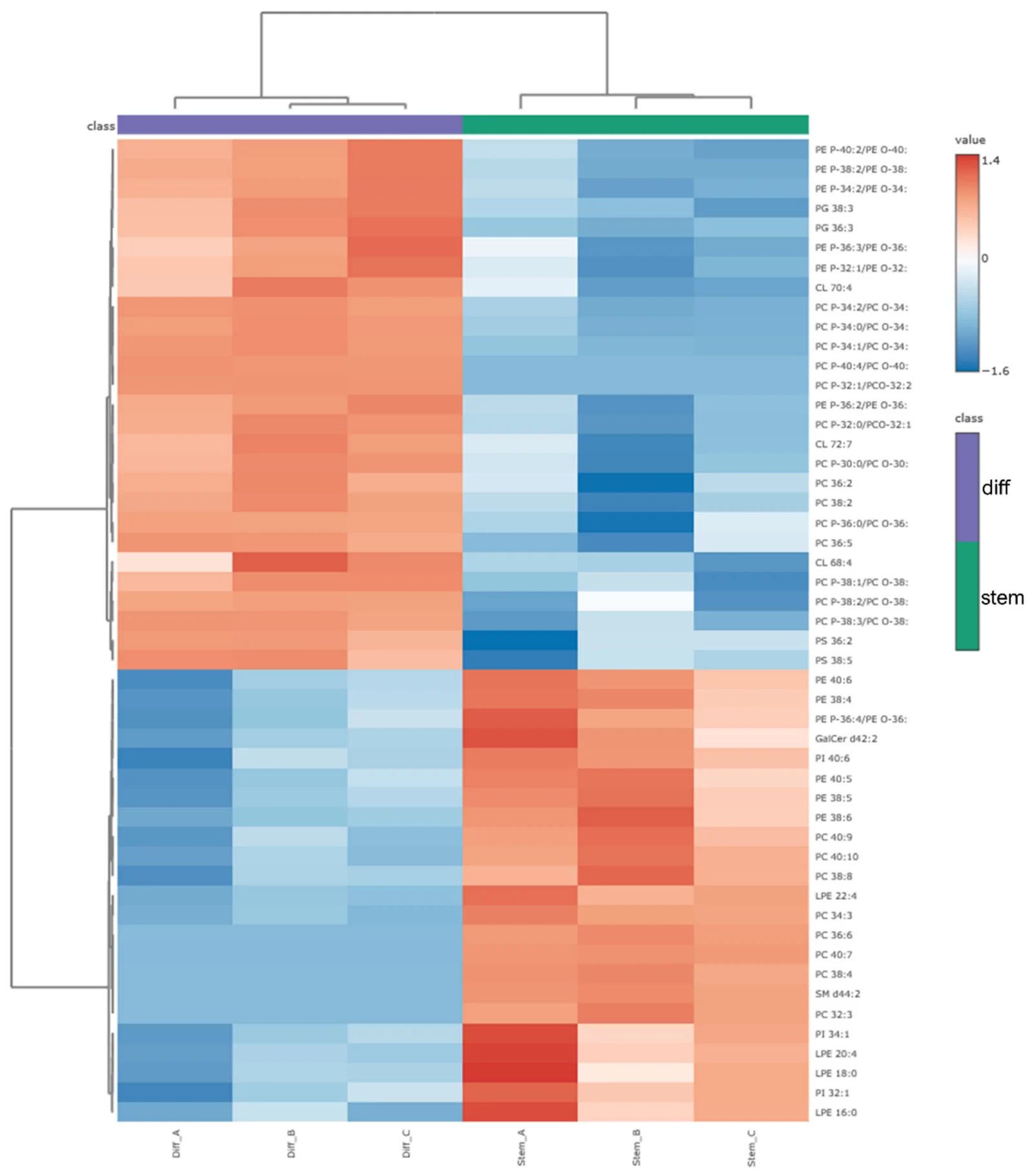
Two-dimensional hierarchical clustering heatmap depicting the presence and relative abundance of lipids in G144 stem (G144-A, G144-B and G144-C) and G144 differentiated (diff-A; diff-B and diff-C) cells. Experimental data are the result of the analysis of 3 independent experiments.

The notable differences observed in the biological behaviour of G144 differentiated cells compared with stem cells, as reported in the previous section, led us to predict a substantial change in the lipid and protein composition of G144 cells upon BMP4-induced differentiation. With respect to the variability of the cell lipidome, the data obtained from the liquid chromatography–mass spectrometry (LC–MS) spectra showed significant differences in the profile of molecular species, within each phospholipid class, between stem and differentiated G144 cells. The identities of phospholipid molecular species, including the fatty acyl composition, as determined on the basis of tandem mass spectrometry (LC–MS/MS) spectra interpretation are listed in Table S1, while the results of the semi-quantification of the lipid species detected (the normalised areas of the peaks of the molecular species identified by mass accuracy through hydrophilic interaction liquid chromatography (HILIC)−MS) are presented in Table S2. The lipidomic analysis workflow allowed the identification and quantification of a total of 196 different lipids, of which 188 are common to undifferentiated and differentiated cell groups (Figure 2B; Tables S1 and S2). Semi-quantitative analysis of lipid species detected by HILIC–MS revealed an apparent difference in the overall lipid profiles of G144 cells before and after BMP4 treatment (Figure 2). Given the small number of independent samples (*n* = 3 per group), this pattern could be interpreted as exploratory in nature. Principal component analysis accounted for 79.6% of the total variance, with Principal Component 1 (PC1) contributing mostly to this total variance (51.8%; Figure 2A). The differences between the two cell groups are highlighted in a heatmap with a two-dimensional hierarchical clustering analysis (Figure 3).

**Figure 4:**
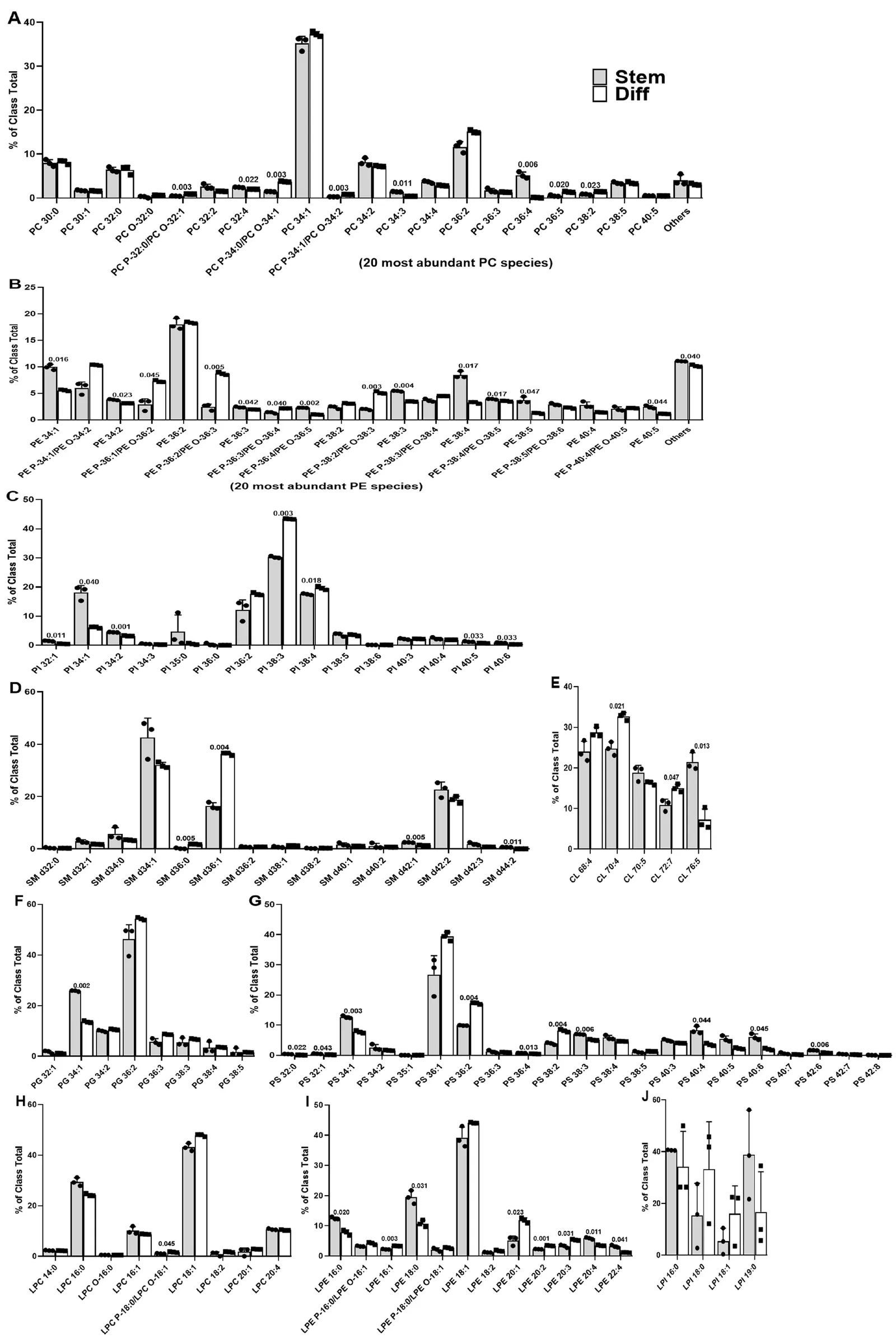
Effects of BMP4 treatment on the relative abundance of individual phospholipid molecular species in G144 stem cells. The relative abundance of the molecular species within each phospholipid class, PC (**A**), PE (**B**), PI (**C**), SM (**D**), CL (**E**), PG (**F**), PS (**G**), and within each lysophospholipid class, LPC (**H**), LPE (**I**), and LPI (**J**). Analysis was performed by HPLC-MS in positive mode, as [M + H]^+^ ions (**A**,**D**,**H**), and in negative mode, as [M − H]^−^ ions (**B**,**C**,**E**–**G**,**I**,**J**). Results are expressed as the percentage contribution of each lipid species within its respective phospholipid class, calculated as its normalised peak area divided by the sum of the normalised peak areas of all species in that class, multiplied by 100. Comparisons between G144 stem cells and differentiated (diff) cells (*n* = 3 independent samples per condition) were performed using two-sided, unpaired Welch’s *t*-tests. The Benjamini–Hochberg procedure was applied across all testable species in the within-class percentage analysis to control the false discovery rate. FDR-adjusted *p* values (q values) are shown on the graph for statistically significant comparisons (q < 0.05). Phospholipid species are reported using sum-composition notation, PL (C), where PL denotes the phospholipid class, C the total number of carbon atoms, and N the total number of double bonds in the constituent chains.

The lipidomic profiling also provided deeper insight into the species variability within each lipid class (Figure 4). The phosphatidylcholine (PC) profile of G144 cells (Figure 4A) clearly shows the predominance of PC 34:1, followed, at a lower concentration, by PC 36:2, in both stem and differentiated cells. An unequal distribution, i.e., higher levels in differentiated cells as compared to stem cells, was noticed among the PC species present at low relative abundance (<5.0%), such as PC 36:5, PC 38:2 and the ether lipids PC P-32:0/PC O-32:1; PC P-34:0/PC O-34:1 and PC P-34:1/PC O-34:2. In contrast, the PC species PC 32:4, PC 34:3, PC 34:4 and PC 36:4 were present at lower levels in differentiated cells compared with stem cells. On the other hand, the PC species 32:3, PC 36:6, PC 38:4 and PC 40:7 were exclusively found in stem cells, whereas the ether lipids PC P-32:1/PC O-32:2 and PC P-40:4/PC O-40:5 were only detected in differentiated cells (Figure 2B).

Regarding the phosphatidylethanolamine (PE) profile (Figure 4B), another important class of lipids in GB cells, the most abundant species is PE 36:2 in both stem and differentiated cells. Other PE species well represented in stem cells are PE 34:1 and PE 38:4, both existing at a significantly higher percentage in these cells as compared to differentiated cells. In the latter cells, the ether lipids PE P-36:1/PE O-36:2, PE P-36:2/PE O-36:3 and PE P-38:2/PE O-38:3 attained levels significantly higher than those observed in stem cells (Figure 4B). Significant differences were also observed within lipid species of the phosphatidylinositol (PI) class, where PI 38:3 is the most abundant species and more prevalent in the BMP4-differentiated cells, with the content in PI 34:1 seemingly compensating for this difference in stem cells (Figure 4C). In the sphingomyelin (SM) class, a noteworthy shift was observed in the most abundant species within the class, changing from SM d34:1 in stem cells to SM d36:1 in the BMP4-differentiated cells (Figure 4D). Significant differences were also observed in cardiolipin (CL) fatty acyl composition between the two groups of cells (Figure 4E). Thus, CL 70:4 (consisting of one palmitoleic acid and three oleic acids, or other combinations containing 16:0 and 18:2) and CL 72:7 (consisting of one palmitoleic acid, two oleic acids and one arachidonic acid, or other combinations containing linoleic acid) present higher levels in differentiated cells than in stem cells. In contrast, the CL species CL 76:5, containing longer fatty acyl chains, including 20 C long (20:0, 20:1, 20:2, 20:3 or 20:4) and 22 C long (22:3, 22:4 or 22:5) fatty acids, presents a content in stem cells that is more than double that in differentiated cells. The fatty acid distribution in the phosphatidylglycerol (PG) class (Figure 4F) seems relatively conserved, with only one species (PG 34:1) differently expressed in the two groups of cells, attaining a higher percentage in stem cells than in differentiated cells. The phosphatidylserine (PS) profile (Figure 4G) revealed a predominance of PS 36:1 in both stem and differentiated cells. Species whose content was higher in differentiated cells than in stem cells are PS 36:2 and PS 38:2. In contrast, the PS species PS 34:1, PS 36:4, PS 38:3, PS 40:4, PS 40:6 and PS 42:6 showed significantly higher levels in stem than in differentiated cells.

Regarding lysophospholipid classes, abundance differences between the two groups of cells were not found for the majority of lysophosphatidylcholine (LPC) species (Figure 4H) and the predominant species of the class LPC as well as of the class LPE, i.e., LPC 18:1 and LPE 18:1 remained unchanged between the two cell groups in terms of percentage within each class (Figure 4H,I). However, most of the other lipids in the LPE class presented significant differences between stem and differentiated cells (Figure 4I). Finally, the content in lysophosphatidylinositol (LPI) was minor and rather variable between samples (Figure 4J).

### 2.3. BMP4-Induced Differentiation of G144 Glioblastoma Stem Cells Promotes Significant Alterations in the Proteomic Pattern

**Figure 5:**
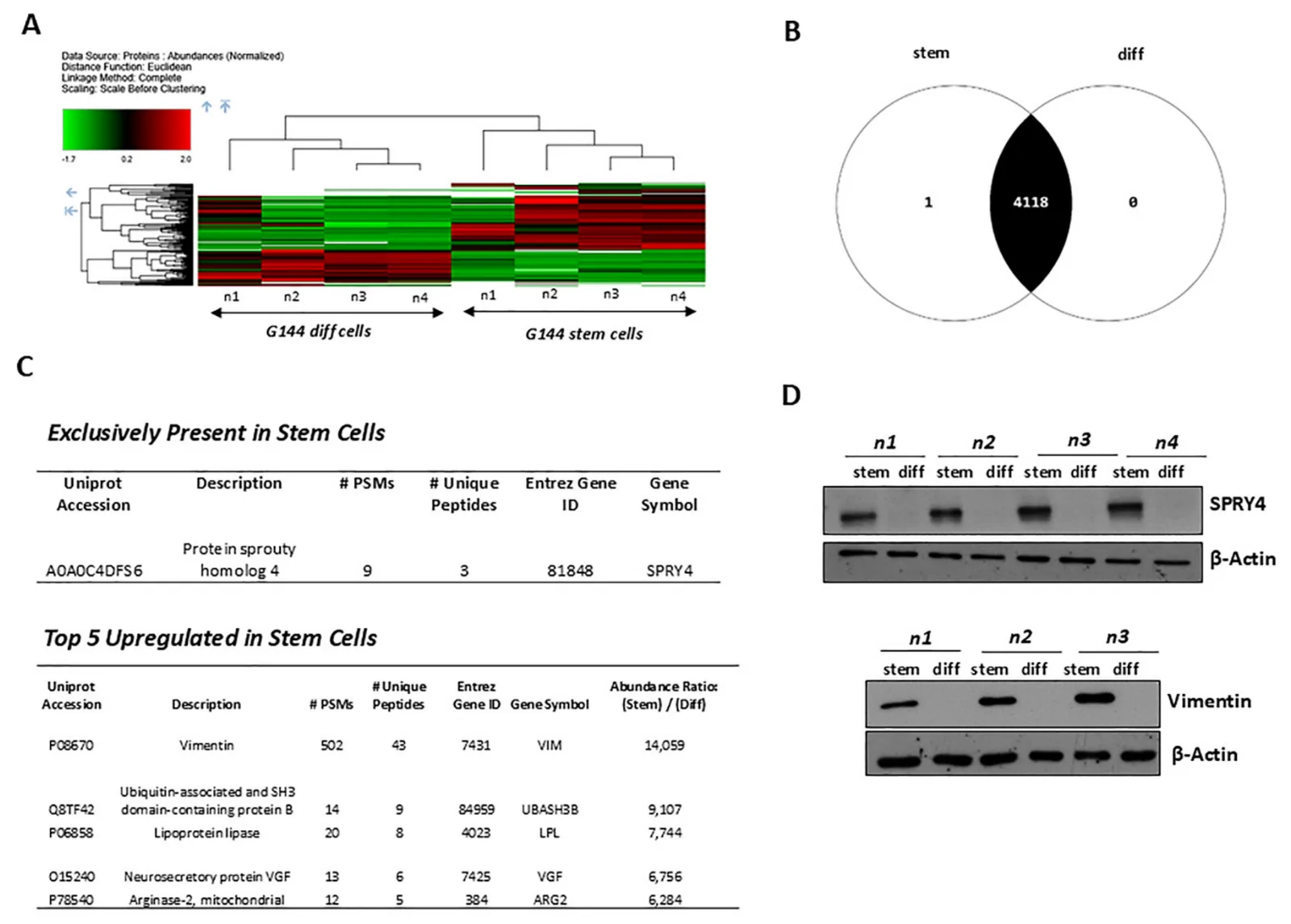
Proteomic analysis of stem and differentiated (diff) G144 cells performed by Liquid chromatography–mass spectrometry (LC–MS/MS). (**A**) Heatmap and (**B**) Venn diagram comparing the relative protein abundance (normalised) of proteins in G144 stem (4 samples) and G144 diff (4 samples) cells. Experimental data are the result of the analysis of four independent experiments. (**C**) Table summarising the protein exclusively present in G144 stem cells and the top 5 upregulated proteins in these cells. (**D**) Expression levels of the protein exclusively present in G144 stem cells (SPRY4) and of the most abundant and upregulated protein in these cells (Vimentin), examined using Western blot analysis. β-actin staining was used as a loading control.

To identify differentially expressed proteins between G144 stem and G144 differentiated cells, proteomic profiles of both cell types were analysed using LC–MS/MS. As shown in the heatmap (Figure 5A), the protein composition of G144 differentiated cells differs markedly from that of G144 stem cells. Importantly, SPRY4 was detected in the G144 stem cells but not detected in the G144 differentiated cells. All the other proteins were present in both G144 stem and G144 differentiated cells at different abundance ratios (Figure 5B). Moreover, proteomic analysis identified several significantly upregulated proteins in G144 stem cells when compared to G144 differentiated cells, with vimentin being the most abundant (Figure 5C). The proteomic data relating to the unique and most abundant proteins present in G144 stem cells were then confirmed by Western blot analysis in at least three different samples (Figure 5D).

In addition, to assess the functional characteristics of G144 stem cells, we used a list of 60 proteins (identified in the proteomic dataset) that were more highly expressed in the G144 stem cells when compared with the G144 differentiated cells (Supplementary Figure S1). WebGestalt enrichment analysis revealed significant overrepresentation of pathways related to DNA methylation and G1-to-S cell cycle control in G144 stem cells compared with G144 differentiated cells. In addition, proteins associated with the retinoblastoma (RB) pathway in cancer and with the activation and regulation of the NRF2 pathway were identified. Analysis of biological processes further revealed enrichment of pathways involved in biological regulation and metabolic processes. Regarding cellular components, the identified proteins were predominantly associated with the nucleus and membrane. In terms of molecular functions, the enriched proteins were mainly involved in protein binding and ion binding.

### 2.4. SPRY4 Silencing in G144 Glioblastoma Stem Cells Results in Higher Expression of the GFAP Astrocytic Differentiation Marker

**Figure 6:**
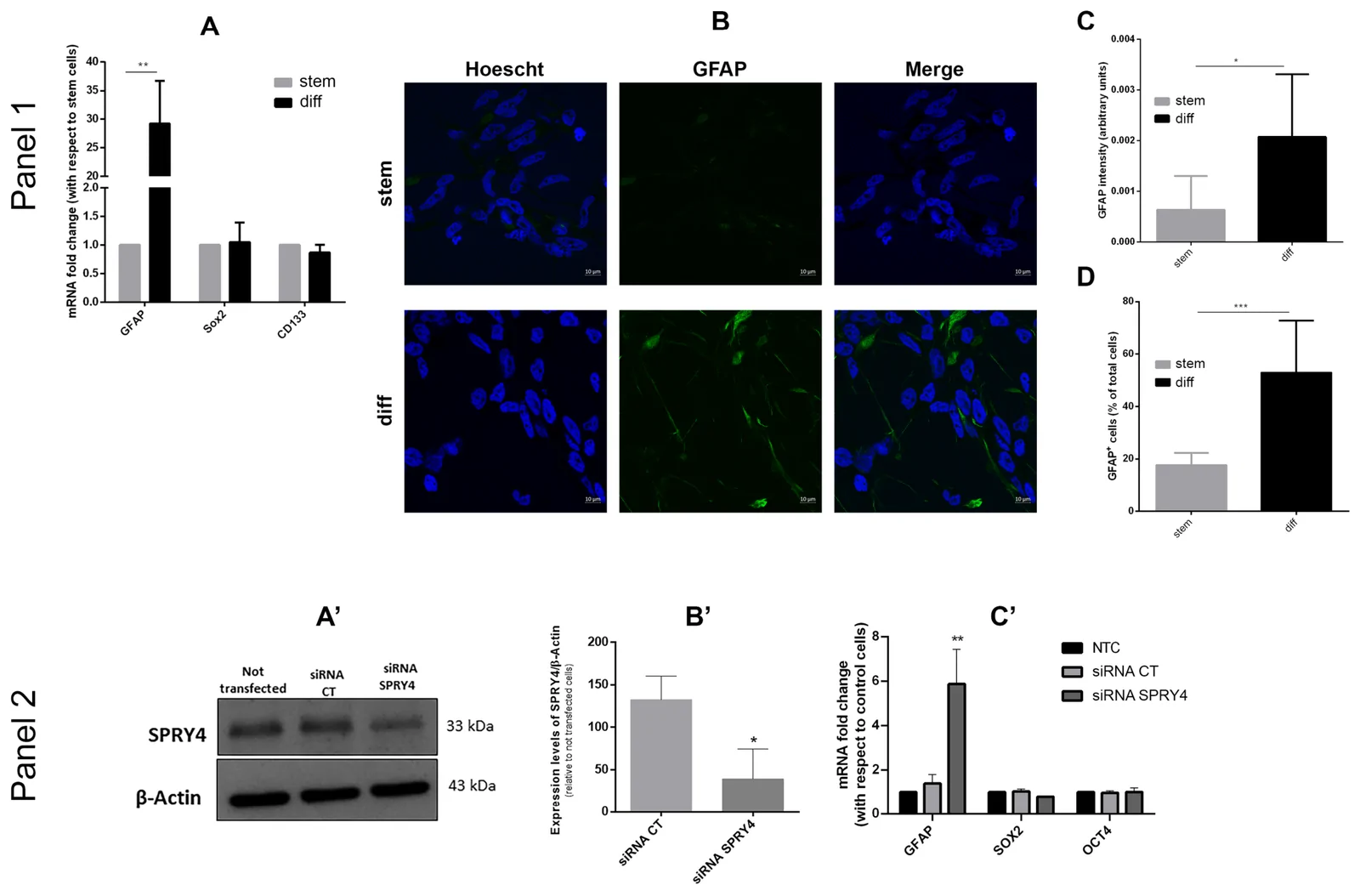
Effects of BMP4-induced differentiation (Panel 1) and of SPRY4 silencing (Panel 2) on the expression of differentiation and stemness markers in G144 cells. mRNA expression levels of GFAP, SOX2, and CD133 were evaluated in G144 stem and differentiated (diff) cells using qRT-PCR and expressed as mRNA fold change with respect to stem cells (**A**). (**B**) shows representative images of GFAP immunocytochemistry (green) (nuclei are labelled with Hoechst, blue). GFAP intensity was determined by analysis of immunocytochemistry images using ImageJ (**C**). The percentage of GFAP-positive cells was determined from immunocytochemistry images, with respect to the total number of cells per field (**D**). Results represent the mean ± SD obtained from 3 independent experiments. Pairwise comparisons were performed between stem and diff cells (* *p* < 0.05, ** *p* < 0.01, *** *p* < 0.001). Expression levels of the SPRY4 protein (**A’**,**B’**) and of mRNA for differentiation and stemness markers (**C’**) were determined in three groups of G144 stem cells: non-transfected (NTC); transfected with a non-targeting control siRNA (siRNA CT); and transfected with a SPRY4-targeting siRNA (siRNA SPRY4). Protein levels of SPRY4 were evaluated by Western blot analysis, and β-Actin was used as a loading control. Expression levels are presented with respect to NTC (**B’**). Results represent the mean ± SD of 3 independent experiments. Pairwise comparisons were performed between cells transfected with a siRNA SPRY4 and with siRNA CT (* *p* < 0.05). mRNA expression levels of GFAP, SOX2, and OCT4 were evaluated by qRT-PCR and expressed as mRNA fold change, with respect to control cells (**C’**). Results represent the mean ± SD of 3 independent experiments. Pairwise comparisons were performed between cells transfected with siRNA SPRY4 and cells transfected with siRNA CT (*p* < 0.01).

As expected, G144 differentiated cells displayed higher expression levels of the GFAP astrocytic differentiation marker than G144 stem cells (Figure 6A–D), although comparable levels of SOX2 or CD133 stemness markers were observed in both cell groups (Figure 6A). In this regard, immunocytochemistry analysis of GFAP, an example of which is shown in Figure 6B, showed that cells cultured in BMP4-containing medium exhibit two-fold higher intensity of GFAP fluorescence (Figure 6C), as well as two-fold more GFAP-positive cells (Figure 6D), than stem cells.

Since SPRY4 protein has been found and validated to be exclusively present in G144 stem cells, i.e., the BMP4-induced cellular differentiation led to the complete loss of SPRY4 detection (as shown in the previous section), we investigated the possibility of forcing the stem cells to differentiate by silencing SPRY4 protein. Figure 6A’,B’ shows that siRNA treatment successfully silenced SPRY4. Upon ensuring that SPRY4 expression could be efficiently downregulated by RNA interference technology, the expression of the astrocytic marker GFAP was analysed in G144 stem cells transfected with a SPRY4-targeting siRNA (siRNA SPRY4) and compared to G144 stem cells treated with a non-targeting control siRNA (siRNA CT) or to non-transfected G144 stem cells (NTC). As observed in Figure 6C’, a significant increase in the levels of the differentiation marker GFAP was found in G144 stem cells upon SPRY4 silencing.

## 3. Discussion

Given the aggressiveness of GB and its associated drug resistance, recent studies have searched for new strategies to make these tumours more sensitive to existing chemotherapeutics. Due to the high genetic heterogeneity of GB, it is predictable that single-target therapies will prove insufficient to combat this disease. On the other hand, it has been shown that a subpopulation of self-renewing cells (GSCs) of GB accounts not only for chemotherapy resistance, but also for tumour recurrence after surgical resection, which is why GSC-targeted therapies hold promise in effectively combating GB [3]. Different strategies for targeting GSCs can be devised, namely those directly affecting their signal pathways and those promoting their differentiation. In this context, previous studies conducted by our research group demonstrated that the differentiation of GSCs induced by a miRNA-based gene therapy approach increased their sensitisation to the action of axitinib, a highly potent anticancer drug, representing an interesting multimodal therapy against GB [15].

Building on previous work, the aim of the present study was to further elucidate the impact of GB stem cell differentiation using the well-established BMP4 treatment strategy in terms of biological effects, namely cell growth, invasion, SA β-gal activity, and tumoursphere formation. In parallel, a lipidomic and proteomic analysis was performed to identify novel therapeutic targets and biomarkers. The discovery of novel therapeutic targets could lead to the development of more targeted and effective pharmaceuticals, or to make GB more responsive to currently used drugs, such as axitinib. On the other hand, lipid- and protein-based biomarkers can be valuable in monitoring disease development, progression, and tumour recurrence, as well as in predicting the vulnerability of tumour cells to therapies. While it is unlikely that a single compositional alteration could constitute a diagnostic or prognostic biomarker, it is plausible to assume that a set of consistently altered cellular protein and lipid components could contribute to identifying the disease and predicting its susceptibility to specific interventions.

Therefore, in this study, we demonstrated that upon BMP4 treatment, which was always coupled with the withdrawal of growth factors from the culture medium, according to the protocol of Pollard and colleagues [7], G144 stem cells showed a decrease in growth rate, enhancement of SA β-gal activity, and reduced invasion and tumoursphere average area. Our results on G144 cell growth corroborate previous reports showing the ability of BMP4 to inhibit the proliferation of human GB stem cells [16]. Our observation of enhanced SA β-gal activity in G144 cells upon BMP4 treatment is also in accordance with previous findings [17]. Interestingly, using different senescence-related assays, Niklasson et al. [17] demonstrated that BMP4 induces p21-dependent senescence in GB, particularly in therapy-resistant mesenchymal-like cells.

Regarding the ability of G144 stem cells to form tumourspheres, our data show that while tumoursphere number remained unchanged upon BMP4 exposure, sphere size (average area) was significantly reduced, consistent with reduced proliferative capacity rather than impaired sphere-forming ability. This pattern aligns with the findings of Sachdeva et al. [11], who showed that BMP4 pathway activation reduces tumoursphere diameter in several GSC lines, including G144 cells, without significantly affecting tumoursphere number. The reduced sphere size is consistent with our observations of decreased G144 cell proliferation and enhancement of a senescence indicator upon BMP4 treatment, suggesting that BMP4 drives G144 cells towards a partially differentiated, quiescent, or senescent state that limits sphere growth. However, other studies using GSC lines established from patient-derived GB primary cultures have reported BMP4-dependent reductions in tumoursphere-forming efficiency, consistent with a partial loss of self-renewal capacity [16,18]. Using a panel of patient-derived GB primary cultures, Verploegh et al. [12] reported that these cultures rarely formed tumourspheres. However, in the subset of cultures that did generate spheres, the sphere number was significantly reduced upon BMP4 treatment. Importantly, by performing single-cell RNA-sequencing analysis, the authors demonstrated that this intervention leads to the loss of stem-like transcriptional clusters and the emergence of differentiated states, thus providing a mechanistic explanation for the impaired sphere-forming capacity observed following BMP4 exposure.

Of note, we also found that exposure of G144 cells to BMP4 significantly decreases invasion in a physiologically relevant 3D culture microenvironment, indicating a shift towards a less aggressive phenotypic behaviour.

Collectively, and consistent with published studies, our findings in G144 glioblastoma stem cells—demonstrating that BMP4 exposure reduces cell proliferation, tumoursphere growth, and invasive capacity while enhancing SA β-gal activity —reinforce the view that BMP4 may act as an effective pro-differentiation trigger with potential therapeutic significance in GB.

It is also relevant to mention here that BMP4 treatment of G144 GSCs promoted a 30-fold increase in the expression of the GFAP astrocytic differentiation marker (Figure 6A–C). However, the stemness markers SOX2 and CD133 (Figure 6A) have not undergone detectable alterations in BMP4-treated cells. Taking all these results together, it should be assumed that differentiated cells, although demonstrating a biological behaviour distinct from stem cells, also differ from typical differentiated cells in several aspects. Therefore, we redefine G144 differentiated cells, obtained in the previously stated culture growth conditions, as cells in a differentiated-like state, reflected by a 30-fold increase in a robust and widely used indicator of astrocytic differentiation (GFAP), as compared to GSCs.

It is important to emphasise that, recently, a prospective, multi-centre, open-label, phase 1 clinical trial was conducted (NCT02869243) to evaluate, for the first time, the safety, feasibility, and efficacy of an intra- and peritumoural administration of human recombinant BMP4 via convection-enhanced delivery in recurrent GB. With this study, it was possible to conclude that BMP4 treatment was safe and well-tolerated, revealing promising therapeutic effects that justify further validation in phase 2 trials [19].

On the other hand, advances in the lipidomics field have allowed the identification of lipids, especially phospholipids (PL), that play an important role in cell homeostasis and signal transduction [20] and have provided significant insight into pathogenic processes, such as cancer. In fact, a well-known cancer hallmark is metabolic reprogramming, affecting not only glucose metabolism [21], but also lipid metabolism [22]. Lipidomic data may, hence, provide important biomarkers for cancer monitoring and reveal new targets for therapeutic interventions, including those involving modulation of specific lipid-metabolising enzymes [23,24,25].

In the present work, an extensive study was performed to identify the plasticity of the phospholipid and sphingolipid classes and species in both G144 stem cells and cells exposed to BMP4. The profile of fatty acid distribution in the principal classes of diacylglycerophospholipids of G144 cells, namely PC, PE, PS, CL, PG and PI, showed high similarities, at least in terms of the most or the two most represented species within each class (Figure 4 A–C,E–G), with that of other well-known glioblastoma cells, such as U87 [25], and cells from GB samples provided by patients undergoing resection [26].

Six lipid species were detected exclusively in G144 stem cells, mostly PCs (PC 32:3; PC 36:6; PC 38:4; PC 40:7), but also a PI species (PI 36:0) and a SM (SM d44:2). In turn, few lipid species were detected exclusively in BMP4 treated cells, namely two ether-lipid PC species (PC P-32:1/PC O-32:2 and PC P-40:4/PC O-40:5; Figure 2B). Overall, BMP4 treatment is associated with an increased abundance of ether-linked lipid species, belonging to both the PC and PE classes, whereas stem cells are characterised by higher levels of several highly unsaturated PCs and PEs, along with a subset of lysophospholipids (specifically LPEs; Figure 3). These differences suggest distinct membrane lipid remodelling patterns associated with the undifferentiated versus differentiated-like cellular states.

The ether lipids, including plasmalogens, whose content was shown to be quite significant in both G144 cell groups, with many of these species being top 20 species in terms of abundance within the class, constitute an important group of membrane lipids in several tissues, such as the brain and heart [27]. These lipids differ from diacylglycerophospholipids in that they have an ether or a vinyl-ether linkage at the *sn*-1 position. The ether lipids that present an ether linkage are designated plasmanyl glycerophospholipids (O-forms), and those containing a vinyl-ether linkage are known as plasmenyl glycerophospholipids or plasmalogens (P-forms). However, our lipidomic analysis could not clearly distinguish between these two groups of lipids, and, therefore, we maintain the broader term “ether lipids”. Although efforts have been made to discriminate the cellular functions of plasmanyl and plasmenyl glycerophospholipids [27], conclusive findings on this issue have not yet been achieved. However, plasmalogens are the most predominant ether lipids in eukaryotic cells and, therefore, the most extensively studied. It has been recognised that plasmalogens exhibit the ability to accommodate in membrane regions with high lipid packing [28,29] and to induce the formation of highly curved membrane structures [28], thus influencing cell membrane remodelling processes [30] and several cellular signalling pathways [27]. In addition, plasmalogens have been proposed as protectors against oxidative stress [27,29,30].

GSCs have been described as highly efficient in maintaining redox homeostasis, in order to preserve their potential for self-renewal and resistance to chemo- and radiotherapies [31]. Therapeutic strategies interfering with the antioxidant defences of GSCs have been attracting increasing interest in clinical settings and pharmacological science [31]. Although the most widely studied of such defences are those related to increased expression of antioxidant enzymes and activation of signalling pathways involving redox-sensitive transcription factors, the role of plasmalogens in preventing ROS cytotoxicity in CSCs should also be considered. Interestingly, the potential of therapeutic strategies targeting plasmalogens has been increasingly explored [32].

Cardiolipin is another class of lipids that has attracted considerable attention in the scientific community as a therapeutic target in various pathologies [33,34], including cancer [35], namely GB [36]. Recently, there has been increasing interest in the mechanisms by which cardiolipin composition and maturity can influence cancer aggressiveness and progress through modulation of mitochondrial functioning. A high diversity of CL species is found in the brain, with an enrichment in long-chain fatty acids such as arachidonic and docosahexaenoic acids, in contrast to CLs from liver and heart, normally enriched in linoleic acid and symmetrical [37]. According to Krieger et al. [38], the characteristics of brain CL patterns are profoundly affected in GB, with a decay in their diversity and the content of long-chain PUFAs, and the appearance of short-chain saturated fatty acids, such as palmitic and stearic acids, indicative of immature CLs. We also observed a relatively narrow range of CL species and a predominance of those possessing short acyl chains, particularly 16:1 and 18:1, in G144 stem cells, both before and after differentiation. However, the longest chain containing CL species (CL 76:5) was the least represented of the 5 most abundant in differentiated cells, but the third most abundant in stem cells, representing a content more than double that in the differentiated cells. The impact of such alteration in mitochondrial functioning deserves to be explored in future work.

As referred to above, another differential characteristic between the lipidome of G144 stem and differentiated cells regards the content of highly unsaturated species. The enrichment in lipids with a high level of unsaturation has been reported for different types of stem cells, namely embryonic, mesenchymal, and pluripotent stem cells, when compared to differentiated cells [39], as well as in cancer stem cells (CSCs), as compared to non-CSCs [40]. In both cases, the enrichment of those lipids has been proposed to contribute to the maintenance of stemness [39].

In general, G144 stem cells were found to be enriched in polyunsaturated species, as compared to differentiated cells. Thus, G144 stem cells showed higher levels of arachidonic acid (*n*-6 C20:4) containing species of PC (PC 36:4), PE (PE 38:4; PE 38:5), and LPE 20:4 than differentiated cells (Figure 4 A,B,I and Supplementary Table S1). Additionally, long-chain polyunsaturated fatty acids were found in higher levels as components of the phospholipids PE (e.g., PE 40:5) and PS (e.g., PS 40:4 and PS 40:6) in stem cells as compared to differentiated cells (Figure 4B,G and Supplementary Table S1). Curiously, phospholipids containing MUFA, such as the oleic acid (C18:1), present higher percentages in differentiated cells as compared to stem cells, e.g., PC 38:2 (predominantly 18:1_20:1); PS 36:1 (18:0_18:1); PS 36:2 (predominantly 18:1/18:1) and PS 38:2 (predominantly 18:1_20:1). Noteworthy, mono and polyunsaturated acyl chains as components of membrane lipids have distinguishable effects on membrane physical properties [41]. Interestingly, Gimple et al. [42] reported that EGFR signalling in glioblastoma stem cells is highly dependent on the synthesis of long-chain PUFAs.

Regarding the proteomic analysis, our results revealed that SPRY4 was detected in the G144 stem cells, but not in the G144 differentiated cells; results were analysed with high statistical confidence across all four samples. This finding is in line with earlier studies reporting that SPRY4 is associated with stemness in mouse embryonic stem cells [43].

The involvement of SPRY4 in carcinogenesis has been investigated across multiple human cancer types, with variable cancer-dependent outcomes. For instance, in human testicular germ cell tumours, SPRY4 knockdown led to decreased cell growth, migration, and invasion, and significantly reduced PI3K/Akt pathway activation, highlighting its role as a possible oncogene in this tumour type [44]. Parallel studies on the knockdown of the long non-coding RNA SPRY4-IT1, a primary transcript derived from SPRY4, indicated that, similarly to SPRY4, it may also exert oncogenic activity in testicular germ cell tumours by activating the PI3K/Akt signalling pathway [44]. Interestingly, some studies have described SPRY4-IT1 as a tumour suppressor in several cancer types, although a growing body of evidence shows that this lncRNA also acts as an oncogene in diverse tissues (reviewed in [45]). As proposed by Ghafouri-Fard et al. [45], SPRY4-IT1 contributes to carcinogenesis possibly through its miRNA-sponge activity, leading to decreased bioavailability of several miRNAs that regulate key signalling pathways involved in oncogenic processes.

Nevertheless, the specific role and functional impact of SPRY4 in GB tumourigenesis remain largely unknown. Recently, the association of SPRY family members (SPRY1-4) with overall and progression-free survival in GB patients was evaluated using public datasets. The authors verified that SPRY1 and SPRY2 may function as oncogenes, whereas the roles of SPRY3 and SPRY4 remain poorly defined [46].

Our proteomic analysis also demonstrated that among the top five upregulated proteins identified in G144 stem cells, vimentin was the most highly expressed when compared with G144 differentiated cells. Vimentin is an intermediate filament protein of the cytoskeleton involved in several structural cellular processes. It is widely established that vimentin is overexpressed during cancer metastasis and has been broadly used as a marker of epithelial-mesenchymal transition (EMT), a process associated with cancer stem cell formation [47]. In fact, EMT-associated transcription factors induce vimentin expression, linking this cytoskeletal protein to increased stemness. In GB, vimentin expression is maintained through reduced proteasomal degradation mediated by deubiquitinase USP7, identified as a major post-translational regulator of protein stability, contributing to the metabolic dysfunction and immune suppression in this tumor type [48]. Therefore, the increased expression of vimentin detected in the G144 stem cells may also contribute to the stem cell properties observed in this cell line.

Moreover, the protein ubiquitin-associated and SH3 domain-containing protein B (UBASH3B) was found to be upregulated in G144 stem cells, as revealed in our proteomic analysis. This protein is known to stabilise receptor tyrosine kinases, particularly the EGFR, by impairing their ubiquitination and degradation, thereby promoting tumour progression [49]. Notably, EGFR amplification, mutation, and overexpression occur in 40-50% of GB cases, driving constitutive activation of the PI3K–AKT–mTOR pathway, which also contributes to GB tumourigenesis [50]. The proteomic analysis further showed that the nerve growth factor-inducible protein (VGF), a neurosecretory protein, is markedly upregulated in G144 stem cells. In fact, VGF expression is known to significantly increase during human brain development [51] and was associated with radio-resistance in prostate cancer, as evidenced by the increased radiosensitivity observed upon siRNA-mediated gene silencing in prostate cancer cell lines [52]. Finally, arginase-2 (ARG2), a mitochondrial enzyme that converts L-arginine into L-ornithine and urea, was also identified among the top five upregulated proteins in G144 stem cells. ARG2 has been implicated in glioblastoma tumourigenesis by promoting the expression of metalloproteinases 2 and 9, which contributes to enhanced cell proliferation, migration and invasion [53]. Altogether, G144 stem cells exhibit malignant features potentially associated with stemness (vimentin), tumour progression (UBASH3B, ARG2), and therapy resistance (VGF), which warrant further investigation in the future.

Interestingly, our enrichment analysis identified pathways related to NRF2 activation and regulation as significantly enriched in G144 stem cells compared with G144 differentiated cells. Indeed, previous studies have demonstrated that SPRY4 can regulate NRF2 expression levels and may therefore be involved in the regulation of inflammation and oxidative stress [54]. Future work should explore the interplay between SPRY4 and NRF2 and the potential involvement of inflammation and oxidative stress in the appearance and maintenance of stemness features in glioblastoma.

Since the SPRY4 protein was expressed in G144 stem cells, but became undetectable after BMP4 treatment, we hypothesised that this protein is involved in the maintenance of stemness and that its downregulation could reinforce or promote *per se* differentiation of G144 cells. Consistent with this hypothesis, we found that RNAi-mediated silencing of SPRY4 in G144 stem cells significantly increased the expression of the astrocytic differentiation marker GFAP. In future work, we will investigate how SPRY4 downregulation affects G144 stem cell biological features (like senescence/viability, proliferation, invasion, and tumoursphere formation) to determine whether its silencing results in changes similar to those observed in the present work with BMP4-differentiated G144 cells.

As mentioned above, contradictory data have been reported regarding the role of SPRY4 across different types of cancer, ranging from oncogenic to tumour-suppressive functions [54], but, collectively, the available evidence emphasises the need to explore its potential as a biomarker and/or therapeutic target. As highlighted by [55] in a recent review, no effective therapy for GB has emerged over the past two decades. This likely reflects not only the heterogeneity of the tumour, but also the possibility that research efforts have not been consistently addressed to the most biologically relevant targets.

One should be aware that the experimental protocol used in this study does not allow for a clear attribution of different omics signatures to stemness and BMP4-induced differentiation of G144 cells. Alternative explanations should be considered, involving the removal of growth factors and its eventual impact on cell proliferation, metabolism, and senescence, as well as reciprocal influences between such events and BMP4 signalling, among others.

However, we believe that our lipidomic and proteomic analysis of G144 stem cells, which identified lipids such as some species of ether lipids, including plasmalogens, polyunsaturated diacylphospholipids and lysophospholipids, and proteins such as SPRY4, may contribute to emphasising the need for a more holistic approach to discover potential biomarkers of stemness and, ultimately, novel molecular targets for GSC-targeted therapies. This is particularly important considering the central role of GSCs in driving tumour heterogeneity, recurrence, and resistance to radiotherapy and chemotherapy. In order to gain more conclusive insights, these studies should be extended to additional GSC models, patient-derived samples, and in vivo models. Additionally, the availability in our laboratory of stable nucleic acid lipid particles (SNALPs), functionalised with the tumour-targeted chlorotoxin peptide, capable of delivering nucleic acids efficiently and specifically to GB, both in vitro and in vivo [56,57], and proved to be internalised by G144 stem cells [15], leads us to believe that we are advancing along a promising path towards more effective strategies to combat this highly aggressive tumour.

## 4. Materials and Methods

### 4.1. Cell Culture and Maintenance

The human GB-derived stem cells, G144, kindly provided by Dr Steve Pollard (University of Edinburgh, Edinburgh, UK), were cultured in Dulbecco’s Modified Eagle’s Medium/Nutrient Mixture F-12 Ham (DMEM/F-12 Ham) (Sigma-Aldrich, Burlington, MA, USA), supplemented with 1.45 g/L D-(+)-glucose (Sigma-Aldrich, Burlington, MA, USA), 1× MEM non-essential amino acids solution (Gibco, Grand Island, NY, USA), bovine serum albumin (BSA), 0.012% fraction V (Gibco), 0.1 mM 2-mercaptoethanol (Gibco), 1% penicillin/streptomycin (Gibco), 0.5× B-27™ Supplement (LifeTech/Gibco, Carlsbad, CA, USA), 0.5× N-2 Supplement (LifeTech/Gibco), which was further freshly supplemented with 10 ng/mL recombinant human FGF-basic (Peprotech, Cranbury, NJ, USA) and 10 ng/mL recombinant murine EGF (Peprotech) (stem culture medium). G144 stem cells were grown adherent in filtered T25 flasks (ThermoFisher Scientific, Waltham, MA, USA) coated with 10 μg/mL laminin (Sigma, St. Louis, MI, USA) and maintained at 37 °C under a humidified atmosphere containing 5% CO_2_. Cells were detached from the flasks upon addition of Accutase Solution (Sigma), followed by the addition of wash medium (DMEM/F-12 supplemented with 1% penicillin/streptomycin and 0.015% BSA) to the obtained cell suspension, which was pelleted by centrifugation at 300 *g* for 4 min at room temperature, as previously described [15].

The G144 cells used in the experiments were between passages 35 and 45. Prior to use, cultures were routinely tested for mycoplasma contamination, and all cultures included in the study were confirmed to be mycoplasma-negative.

G144 stem cells were plated at the appropriate density in stem culture medium onto laminin-coated plates and incubated for 6 days at 37 °C, under a humidified atmosphere containing 5% CO_2_, prior to any experiment [15].

For experiments with G144 differentiated cells, 6 days after G144 stem cell plating, the stem culture medium was replaced with differentiation culture medium (DMEM/F-12 complete medium supplemented with 10 ng/mL BMP4 (Peprotech) and lacking recombinant human FGF-basic and recombinant murine EGF). Cells were incubated for 7 days at 37 °C under a humidified atmosphere containing 5% CO_2_ prior to any experiment.

### 4.2. Assessment of Cell Proliferation

Proliferation of G144 stem and differentiated cells was evaluated using the EdU incorporation assay for detection of DNA synthesis [24] and the SRB assay for determination of the cellular protein content [24,58]. Briefly, for the EdU incorporation assay, stem and differentiated cells were plated in the appropriate culture medium (stem or differentiated medium) onto 12-well plates containing coverslips coated with laminin, and EdU (Lumiprobe GmbH, Hannover, Germany) was added to the medium. Twenty-four hours later, cells were washed with phosphate-buffered saline (PBS), fixed with 4% PFA in PBS, permeated with 0.2% Triton X-100 in PBS, further washed with PBS, and incubated with a solution of Sulfo-Cyanine3 azide (Lumiprobe), CuSO_4_ (Sigma), and ascorbic acid (Sigma) in the dark, at room temperature, for 30 min. After this period of incubation, cells were thoroughly washed in PBS, and nuclei were stained with Hoechst 33342 dye (Molecular Probes). Cells were then observed under a Carl Zeiss LSM 710 confocal microscope (Zeiss, Jena, Germany), and EDU-positive stem and differentiated cells were counted and presented as a percentage of the total number of each type of cell in each field, as determined with Hoechst nuclei labelling.

Briefly, for SRB protein content determination, both stem and differentiated cells were seeded in the appropriate culture medium onto 96-well plates, fixed with 1% acetic acid in methanol at different time points (days 0, 2, 4, 7 and 10), starting at t = 0, corresponding to the plating day, and stored at −20 °C for 1 h. The solution was then removed; the cells were dried at 37 °C and incubated with 0.5% SRB solution containing 1% acetic acid in water for 1 h at 37 °C. Subsequently, the cells were washed with a solution of 1% acetic acid in water and dried at 37 °C. Protein-bound SRB was solubilised with 10 mM Tris base buffer (pH = 10), followed by incubation for 2 h under mild agitation in the dark. Absorbance was measured at 510 nm in a SpectraMax Plus 384 (Molecular Devices, San Jose, CA, USA). Stem and differentiated cell density at each time point, which was taken as proportional to protein content, was expressed with respect to t = 0.

### 4.3. Evaluation of Spheroid Growth and Cell Invasion

For the cell invasion analysis, G144 stem and differentiated cells were grown into three-dimensional spheroids in Corning Costar Ultra-Low Attachment (ULA) 96-well plates with round bottom. For spheroid formation, cells were allowed to grow for 7 days at 37 °C under a humidified atmosphere containing 5% CO_2_, as described before [59]. Growth of spheroids, produced from both stem and differentiated cells, was evaluated by taking pictures over time for 13 days, using a Celena Digital Imaging System (Logos Biosystems, Annandale, VA, USA). The cell invasion assay was performed as described before [59]. Briefly, spheroids produced from stem or differentiated cells were embedded into a collagen matrix, prepared by gently mixing PureCol bovine collagen type I solution (Advanced BioMatrix, Carlsbad, CA, USA) with Minimum Essential Medium (10×) and 0.1 M (5×) NaOH, to achieve a final collagen concentration of 2.2 mg/mL in 24-well plates. After polymerisation initiation, stem or differentiation medium was added on top of the collagen matrix. Three days after collagen embedding, cell invasion was monitored by digital photography in a Carl Zeiss Axio Observer Z1 microscope equipped with a CCD digital camera (AxiocamHRm), using a 4×/0.10 objective. Invasion areas for spheroids produced from stem and differentiated cells were quantified using the Image J software (v. 1.48, Wayne Rasband, NIH, Bethesda, MD, USA).

### 4.4. Evaluation of a Cell Senescence Indicator

The β-galactosidase activity assay was used, as described before [15], as an indicator of senescence of both G144 stem and differentiated cells. Briefly, stem and differentiated cells were plated at a density of 18 000 cells/well in the appropriate culture medium (stem or differentiation medium) onto 12-well plates containing coverslips coated with laminin. After being washed with PBS and fixed with 4% PFA for 15 min at room temperature, cells were further washed and stained with staining solution (40 mM citric acid, 40 mM sodium phosphate, 5 mM K_3_Fe[CN]_6_, 5 mM K_4_Fe[CN]_6_, 2 mM MgCl_2_, 150 mM NaCl, 1 mg/mL X-Gal in DMSO, pH 6.0) overnight in the dark, at 37 °C, in a non-CO_2_ incubator. Cells were washed with PBS, incubated with 2 μg/mL Hoechst 33342 for 5 min, washed again, and the coverslips were mounted on glass slides with DAKO fluorescence mounting medium (Agilent Technologies, Santa Clara, CA, USA) and allowed to cure overnight at room temperature. Fluorescence/colour microscopy images of stem and differentiated cells were taken with a 20× objective using an Axio Imager Z2 widefield microscope, equipped with a CCD monochromatic digital camera (Axiocam HRm) and a CCD colour digital camera (Axiocam HRc), and the coupled software ZEN Pro (Zeiss, Germany). Three images were taken for each condition.

### 4.5. Assessment of Cell Ability for Tumoursphere Formation

G144 stem and differentiated cells were plated at a density of 5000 cells/well onto 48-well plates (ThermoFisher Scientific), coated with 1% agarose to prevent cells from adhering to the plate, in stem culture medium (complete DMEM/F-12 supplemented with rhFGF and rmEGF) for stem cells or in differentiated culture medium (complete DMEM/F-12 supplemented with BMP4) for differentiated cells. Four days after plating, the cells’ ability to form tumourspheres was assessed in terms of the number and area of the tumourspheres by transmission microscopy, with images of the entire well taken in tiles with a 10× objective in an Axio Observer Z1 inverted widefield microscope, equipped with a CCD digital camera (Axiocam HRm) and controlled by the ZEN Blue software. The number and the area of the tumourspheres were determined using the ImageJ software (v. 1.48, Wayne Rasband, NIH, USA), as previously described [15].

### 4.6. Lipid Extraction and Quantification of Total Phospholipid

Cell pellets were resuspended in 1 mL of ice-cold distilled water and subjected to sonication. Small aliquots (4 μL) were collected for protein determination using the DC Protein Assay (Bio-Rad, Hercules, CA, USA). Total lipid extraction was subsequently performed according to the method of Bligh and Dyer, as previously described [60]. An aliquot of the resulting lipid extract (50 μL) was transferred into glass tubes and evaporated to dryness at room temperature. Total phospholipid content was then determined through a phosphorus-based colorimetric modified version of the Bartlett and Lewis method [61]. Briefly, 0.163 mL of 70% (*v*/*v*) perchloric acid was added to the dried lipid residue, and samples were digested at 180 °C for 2 h using a heating block, with each tube loosely covered by a glass marble to prevent evaporation losses. Following digestion, 0.825 mL of distilled water, 0.125 mL of 2.5% (*w*/*v*) ammonium molybdate, and 0.125 mL of freshly prepared 10% (*w*/*v*) ascorbic acid were sequentially added. The reaction mixture was then incubated in a boiling water bath for 10 min, after which absorbance was measured at 800 nm using a microplate reader (SpectraMax Plus 384). Phosphorus content was quantified using a calibration curve generated from a phosphorus standard solution (Sigma), covering a range of 3.25 to 156 nmol of phosphorus and processed in parallel with the samples.

### 4.7. Hydrophilic Interaction Liquid Chromatography–Mass Spectrometry (HILIC-LC–MS)

The characterisation of the polar lipidome in the total lipid extracts was performed by hydrophilic interaction liquid chromatography on a high-performance liquid chromatography (HPLC) Ultimate 3000 Dionex (Thermo Fisher Scientific, Bremen, Germany), with an autosampler coupled online to a Q-Exactive hybrid quadrupole mass spectrometer (Thermo Fisher, Scientific, Bremen, Germany). The solvent system used consisted of two mobile phases: mobile phase A (water, acetonitrile, and methanol, 25:50:25) containing 5 mM ammonium acetate and mobile phase B (acetonitrile and methanol, 60:40) containing 5 mM ammonium acetate.

Elution was set to start at 5% of mobile phase A, held isocratically for 2 min, followed by a linear increase to 65% of this phase within 13 min, after which this value was maintained for 2 min, and later returned to the initial settings within 30 min (3 min for the decrease to the initial 10% of phase A, and a re-equilibration period of 10 min prior to the next injection). For HILIC-LC–MS analysis, an aliquot of 10 µg of quantified phospholipid, 8 µL of phospholipid standards mix (dMPC—0.01 µg, dMPE—0.01 µg, LPC—0.01 µg, dPPI—0.04 µg, dMPG—0.006 µg, dMPS—0.02 µg, tMCL—0.04 µg, SM(17:0/d18:1)—0.01 µg, dMPA—0.04 µg) and 82 µL of eluent (5% of mobile phase A and 95% of mobile phase B) were mixed and a volume of 10 μL of the mixture injected into an Ascentis Express HILIC column (10 cm × 2.1 mm × 2.7 µm, Supelco, Sigma-Aldrich), with a flow rate of 50 µL min^−1^ at 35 °C. Data acquisition was performed in both positive (electrospray voltage 3.0 kV) and negative (electrospray voltage −2.7 kV) modes, with a high resolution of 70,000 and an AGC target of 1e6. The capillary temperature was 250 °C, and the sheath gas flow was 15 U. The MS/MS determinations were performed with a resolution of 17,500 and an AGC target of 1 × 10^5^. Cycles consisted of one full scan mass spectrum, and ten data-dependent MS/MS scans were repeated continuously throughout the experiments, with a dynamic exclusion of 60 s and an intensity threshold of 1 × 10^4^. Normalised collision energy (CE) ranged between 25, 30, and 35 eV. Data acquisition was performed with the Xcalibur data system (V3.3, Thermo Fisher Scientific, USA). Molecular lipid species were annotated using Lipostar software (Molecular Discovery Ltd., Borehamwood, UK, version 2.1.5) [62], which was used for raw data import, peak detection, integration, and annotation. Lipid assignments were searched against a database generated from the LIPID MAPS structure database (June 2024 version) and fragmented using the Lipostar DB Manager Module according to the software’s fragmentation rules. Automatic peak picking was performed with the SDA smoothing level set to high and a minimum signal-to-noise ratio of 3. Isotope clustering used a mass tolerance of 7 ppm and a retention time tolerance of 0.2 min.

Lipid species were annotated when the precursor ion mass error was below 5 ppm. Where MS/MS spectra were available, product ions were evaluated using a mass tolerance of 10 ppm, and the proposed annotations were manually checked against the corresponding spectra. Annotations supported only by accurate precursor mass were distinguished from those supported by MS/MS evidence. Peak areas were normalised to the area of the corresponding lipid-class internal standard added at a known concentration. The relative abundance of each species within its lipid class was calculated by dividing its normalised peak area by the sum of the normalised peak areas of all species assigned to that class.

A zero in the peak-area table indicates that no qualifying peak area was reported for that sample; it does not demonstrate biological absence. Zeros were retained in the tabulated data and univariate analysis. For PCA in MetaboAnalyst, zero values were replaced with half the smallest non-zero normalised peak area according to the preprocessing settings used.

### 4.8. Proteomic Analysis Using Liquid Chromatography–Mass Spectrometry/Mass Spectrometry (LC–MS/MS)

Protein samples (20 µg) from four independent experiments were analysed using liquid chromatography with tandem mass spectrometry (LC–MS/MS). Samples were processed using a solid-phase-enhanced sample preparation (SP3) procedure, followed by enzymatic digestion with Trypsin/LysC. Protein identification and quantitation were performed by nano LC–MS/MS, according to a previously published protocol [63,64]. Data analysis was processed using Proteome Discoverer 3.3.0.386 software (Thermo Scientific) and searched against the UniProt database for the *Homo sapiens* proteome and a common contaminant database. Protein-label-free quantitation was performed with the Minora feature detector node using the following parameters: peptides to use unique plus razor, precursor abundance was based on intensity, normalisation mode based on total peptide amount, pairwise protein ratio calculation, and hypothesis tested using *t*-test (background-based). The LC–MS/MS procedures and raw-data processing were performed by the Proteomics Scientific Platform, i3S, Porto, Portugal.

The protein content of G144 stem cells and G144 differentiated cells was analysed in four independent biological replicates. The results, presented as the average of four independent experiments, were analysed according to several predefined criteria. Proteins identified in the *Bos taurus* database were excluded, and the dataset was filtered according to the following parameters: “unique peptides” greater than or equal to 2, “razor peptides” greater than or equal to 2, an abundance ratio greater than or equal to 2, and a *p*-value less than or equal to 0.05. Only proteins identified with a confidence level of at least “High” in Proteome Discoverer and consistently detected across all four biological replicates were included in the analysis. To identify proteins exclusively expressed in the G144 stem cells, proteins had to be detected in all four G144 stem cell samples and absent from all four G144 differentiated cell samples. The mass spectrometry proteomics data have been deposited in the ProteomeXchange Consortium via the PRIDE partner repository (identifier PXD083664). The raw data (referring to all proteins excluding protein contaminants and proteins present in the *Bos taurus* database) is shown in Supplementary Table S3.

Furthermore, functional enrichment analysis was performed using WebGestalt by uploading a list of 60 proteins that were more highly expressed in the G144 stem cells when compared with the G144 differentiated cells.

### 4.9. Protein Extraction and Western Blot Analysis

Cell pellets obtained from both G144 stem and differentiated cells were lysed using Winman’s buffer (1% NP-40, 0.1 M Tris-HCl pH 8.0, 0.15 M NaCl, and 5 mM EDTA) supplemented with EDTA-free protease inhibitor cocktail (Roche) and phosphatase inhibitor (Sigma), with agitation for 30 min at 4 °C [63]. After centrifugation (13 000 rpm, 10 min, 4 °C), protein lysates were quantified using a modified version of the Lowry assay (DC™ Protein Assay reagents, Bio-Rad; 5000116). A total of 20 μg of protein was separated by SDS-PAGE and transferred onto a nitrocellulose membrane. Membranes were stained with Ponceau S solution to confirm protein transfer. The membranes were blocked in Tris-buffered saline solution with 0.1% Tween-20 containing 5% (*w*/*v*) non-fat dry milk at room temperature for at least 1 h, under agitation, and then incubated overnight, under agitation at 4 °C, with the following primary antibodies: anti-SPRY4 (1:500) polyclonal antibodies (Invitrogen, Waltham, MA, USA); anti-Vimentin sc-6260 (1:500) and anti-β-Actin sc-47778 (1:2000) (Santa Cruz Biotechnology, Dallas, TX, USA). Afterward, a secondary antibody was added, followed by 1 h incubation at room temperature with agitation. The membrane signal was detected using the enhanced chemiluminescence (ECL) Western blot detection Reagent (Bio-Rad), the Amersham Hyperfilm ECL (GE Healthcare, Chicago, IL, USA), and the Kodak GBX developer and fixer (Sigma). The intensity of the bands was analysed using Quantity One 1-D Analysis software (Bio-Rad).

### 4.10. Cell Transfection

The siRNA duplexes targeting SPRY4 (siRNA SPRY4; sense 5′ CCA AGG UGG UUC ACU GUA AdTdT 3′) and the non-targeting siRNA duplex (siNT CT; sense 5′ UUC UCC GAA CGU GUC ACG UdTdT 3′) were synthesised by GeneCust (Ellange, Luxembourg).

Complexes of siRNA with Lipofectamine RNAiMAX (Life Technologies, Carlsbad, CA, USA) were prepared using 1 μL of transfection reagent per 20 pmol of siRNA in serum-free Opti-MEM, gently mixed, and left to incubate for 20 min at room temperature to allow for the formation of complexes. Cells (90 000 cell/well) were incubated with the complexes at the final siRNA concentration of 100 nM per well. After 4 h, the medium was aspirated and fresh complete medium was added to the wells. Plates were then incubated for 72 h at 37 °C under a humidified atmosphere containing 5% CO_2_.

### 4.11. Quantification of mRNA Levels by qRT-PCR

RNA of G144 cells was extracted using Zymo Quick-RNA Miniprep Kit (Zymo Research, CA, USA) and quantified using the NanoDrop 2000 Spectrophotometer (ThermoScientific). The reverse transcription for mRNA quantification of both G144 stem and differentiated cells was performed using the NZY First-Strand cDNA Synthesis Kit (NZYtech, Lisbon, Portugal. The protocols used followed the manufacturer’s specified guidelines.

The mRNA levels of the differentiation (GFAP) and stemness (SOX2, CD133 and OCT4) markers for G144 stem and differentiated cells were determined by real-time PCR (qRT-PCR) in a StepOnePlus Thermocycler (Applied Biosystems), using 96-well microtiter plates and the SsoAdvanced Universal SYBR Green Supermix (Bio-Rad), as described before [15]. The primers for GFAP, SOX2, CD133, and OCT4 genes and the reference gene (HPRT1) were predesigned by Qiagen (QuantiTect Primer, Qiagen, Hilden, Germany). Threshold values for threshold cycle determination (Ct) were set to 10 000, and the baseline was determined automatically by the StepOne Software (V. 2.3). Fold change of mRNA levels in differentiated cells with respect to stem cells or in siRNA-transfected stem cells with respect to control cells was determined by the Pfaffl method, taking into consideration the different primer amplification efficiency of each gene in all experiments. The amplification efficiency for each marker gene or reference gene was determined according to the equation: E = 10^−1/S^, where S is the slope of the obtained standard curve.

### 4.12. Protein Detection by Immunocytochemistry

GFAP immunocytochemistry experiments were performed, as described before [15]. Briefly, G144 stem and differentiated cells, plated at a density of 18 000 cells/well in the appropriate culture medium (stem or differentiation medium) onto 12-well MW plates containing coverslips coated with laminin, were washed with ice-cold PBS and fixed with 4% paraformaldehyde for 15 min at room temperature. After fixation, the cells were further washed and blocked in blocking buffer (PBS/3% BSA/0.3% Triton X-100) for 1 h at room temperature. Cells were subsequently incubated with the primary GFAP antibody (1:1000) overnight at 4 °C, washed with PBS, and incubated with the FITC-conjugated secondary antibody (1:500) for 2 h. Cells were further washed, incubated with 2 µg/mL Hoechst 33342 dye for 5 min, washed again, and coverslips were mounted on slides with DAKO fluorescence mounting medium (Agilent Technologies, Santa Clara, CA, USA) and allowed to cure overnight at room temperature. Fluorescence microscopy images of stem and differentiated cells were taken with a 40× objective using a Carl Zeiss LSM 710 confocal microscope (Zeiss, Germany) and the coupled software ZEN Pro (Zeiss, Germany). GFAP intensity was determined using ImageJ software (v. 1.48, Wayne Rasband, NIH, USA). GFAP-positive stem and differentiated cells were counted in each field and are presented with respect to the total number of each cell type in each field, as determined by the total number of nuclei in each field. At least three microscopic fields were analysed per independent experiment. Images were assessed systematically using consistent criteria across experimental conditions, with the aim of minimising potential bias.

### 4.13. Statistical Analysis

Results from experiments on the cell biological behaviour are presented as mean ± SD of at least three independent experiments performed in duplicate.

Data were analysed using GraphPad Software (La Jolla, CA, USA) and statistical significance was set at *p* < 0.05.

For lipidomic analysis, three independent samples were analysed per condition. Differences between G144 stem cells and BMP4-differentiated G144 cells were assessed using two-sided, unpaired Welch’s t-tests for both normalised peak areas and the percentage contribution of each lipid species within its respective class. The Benjamini–Hochberg procedure was applied separately across the 196 species tested using normalised peak areas and the 194 testable species analysed using within-class percentages. FDR-adjusted *p* values (q values) < 0.05 were considered statistically significant. Fold changes were calculated as the mean value in differentiated cells divided by the mean value in stem cells. Zero values were retained in the univariate analyses. Individual sample values, means, fold changes, unadjusted *p* values, and q values are reported in Supplementary Table S2. Graphs depicting the results of the analyses are presented as the mean ± standard deviation (SD) of at least three independent experiments and were produced using GraphPad Prism 8.0 (GraphPad Software, La Jolla, CA, USA).

Principal component analysis and hierarchical clustering were performed in MetaboAnalyst (http://www.metaboanalyst.ca/; accessed on 1 May 2026) using normalised peak areas. For these analyses, zero values were replaced with half the smallest non-zero normalised peak area according to the preprocessing settings used. A 50% missing-value threshold was set, with no additional feature filtering. Data were log10-transformed and autoscaled by mean-centreing and division by the SD of each variable. Given the three independent samples per condition, the multivariate analyses were interpreted as exploratory.

## Data Availability

The mass spectrometry proteomics data has been deposited in the ProteomeXchange Consortium via the PRIDE partner repository with the dataset identifier PXD083664.

## Supplementary Materials

The following supporting information can be downloaded at: https://www.mdpi.com/article/10.3390/ijms27219517/s1.
