# Supplementary material for: Differentiation of Human Glioblastoma Stem Cells as a Prospective Tool to Find New Biomarkers and Molecular Targets: A Proteomic and Lipidomic Approach
Source: Int J Mol Sci. 2026 Oct 24;27(21):9517. doi: 10.3390/ijms27219517 (PMC13762967; doi:10.3390/ijms27219517)
Supplement: Supplementary file 1 [file ijms-27-09517-s001.zip › Supplementary Figure S1.pdf]

Supplementary Figures

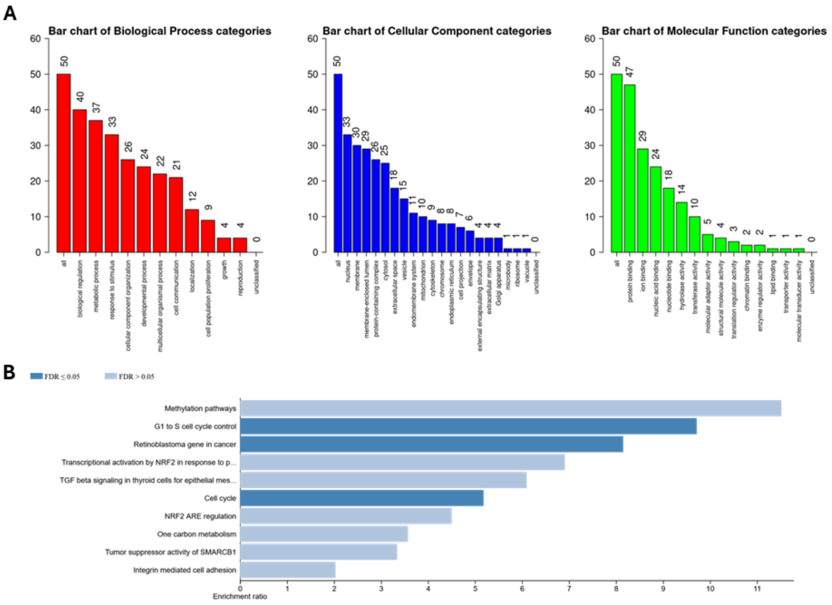

Supplementary Figure S1: Functional analysis of 60 proteins more highly expressed in G144 stem cells compared with G144 differentiated cells, from the proteomic dataset, analyzed using WebGestalt (WEB-based GEne SeT AnaLysis Toolkit). A) Enrichment analysis for biological processes (red), cellular components (blue), and molecular functions (green). B) Overrepresentation Enrichment Analysis (ORA) showing enriched functional categories in G144 stem cells, colored according to the False Discovery Rate (FDR) threshold (FDR ≤ 0.05 and FDR > 0.05).
